# Improving Communication at an In-Patient Female Forensic Intellectual Disability Unit – Delamere Ward

**DOI:** 10.1192/bjo.2024.432

**Published:** 2024-08-01

**Authors:** Shelton Sibanda, Claire Lloyd, Nathan Hunter, Abigail Williamson

**Affiliations:** Merseycare NHS Foundation Trust, Liverpool, United Kingdom

## Abstract

**Aims:**

To improve communication in a Female Forensic Intellectual Disability ward.

**Methods:**

Delamere ward is a medium secure Intellectual Disability female forensic ward in Merseycare NHS Foundation Trust. Admitted patients have varying levels of need and complexities. A Multidisciplinary Team (MDT) comprising of Medical, Nursing, Support workers, Psychology, Occupational therapists and Speech and Language therapists, among others, works with patients in this ward. The MDT meets regularly in various patient meetings with decisions taken communicated to patients and staff through existing means. Communication was noted to be ineffective, leading to patient frequent challenging behaviours and patient dissatisfaction.

The Quality Improvement project was registered with the local Trust Quality Improvement Department at the outset. Staff views on ward communication effectiveness were gathered using entrance and exit questionnaires. 12 and 9 staff members responded to the entrance and exit questionnaires respectively.

The project was conducted over a period of 12 weeks and was divided into Service user and Staff led initiatives. Service user initiatives focused on strengthening existing community meetings, use of ward health promotion boards, MDT walk arounds and utilisation of ward areas. Staff initiatives included introduction of daily morning handover meetings, strengthening of existing staff meetings, listening sessions with staff, and use of reflective sessions. Daily handover meetings were open to the whole ward team and attended per staff availability. Ward dynamics encompassing the previous day were discussed and documented. Qualitative staff views transcribed and compared pre and post project.

**Results:**

Implementation of the quality improvement project eased tension between the MDT and the wider team, helped foster more shared decision making, increased team participation, bridged the gap between fortnightly held ward rounds, created a platform for prompt information sharing, encouraged bidirectional flow of information and helped therapists plan their sessions accordingly.

**Conclusion:**

Effective ward communication was beneficial to staff and patients alike, leading to better implementation of care plans, increased staff confidence and teamwork, and service user satisfaction.

